# InterVA versus Spectrum: how comparable are they in estimating AIDS mortality patterns in Nairobi's informal settlements?

**DOI:** 10.3402/gha.v6i0.21638

**Published:** 2013-10-24

**Authors:** Samuel Oji Oti, Marilyn Wamukoya, Mary Mahy, Catherine Kyobutungi

**Affiliations:** 1 African Population and Health Research Center, Nairobi, Kenya; 2 Department of Global Health, Amsterdam Institute for Global Health and Development, Academic Medical Center, University of Amsterdam, Amsterdam, The Netherlands; 3 UNAIDS, Geneva, Switzerland

**Keywords:** AIDS, InterVA, Spectrum, mortality, Nairobi

## Abstract

**Background:**

The Spectrum computer package is used to generate national AIDS mortality estimates in settings where vital registration systems are lacking. Similarly, InterVA-4 (the latest version of the InterVA programme) is used to estimate cause-of-mortality data in countries where cause-specific mortality data are not available.

**Objective:**

This study aims to compare trends in adult AIDS-related mortality estimated by Spectrum with trends from the InterVA-4 programme applied to data from a Health and Demographic Surveillance System (HDSS) in Nairobi, Kenya.

**Design:**

A Spectrum model was generated for the city of Nairobi based on HIV prevalence data for Nairobi and national antiretroviral therapy coverage, underlying mortality, and migration assumptions. We then used data, generated through verbal autopsies, on 1,799 deaths that occurred in the HDSS area from 2003 to 2010 among adults aged 15–59. These data were then entered into InterVA-4 to estimate causes of death using probabilistic modelling. Estimates of AIDS-related mortality rates and all-cause mortality rates from Spectrum and InterVA-4 were compared and presented as annualised trends.

**Results:**

Spectrum estimated that HIV prevalence in Nairobi was 7%, while the HDSS site measured 12% in 2010. Despite this difference, Spectrum estimated higher levels of AIDS-related mortality. Between 2003 and 2010, the proportion of AIDS-related mortality in Nairobi decreased from 63 to 40% according to Spectrum and from 25 to 16% according to InterVA. The net AIDS-related mortality in Spectrum was closer to the combined mortality rates when AIDS and tuberculosis (TB) deaths were included for InterVA-4.

**Conclusion:**

Overall trends in AIDS-related deaths from both methods were similar, although the values were closer when TB deaths were included in InterVA. InterVA-4 might not accurately differentiate between TB and AIDS deaths.

It appears that the global fight against AIDS is bearing positive results in most countries burdened by the epidemic (1). New infections and AIDS deaths have declined significantly over the last decade (1). In Kenya, the annual number of adult new infections dropped from 102,000 in 2000 to 91,000 in 2011, and the number of adult AIDS deaths has reduced significantly within the same period (2).

However, in the face of persistent global economic uncertainty, there are doubts as to whether the continued global investments in the HIV response can be sustained (3–5). As of 2011, there were over 1.4 million adults living with HIV in Kenya. About 592,000 adults were eligible for antiretroviral therapy (ART), and about 83% of those received ART (6).

To ensure continued funding, countries need to generate empirical evidence that demonstrates their progress in the HIV response. Population-level impact data will be particularly useful as many HIV programmes have been in existence for several years. However, many of the most affected countries lack the complete vital registration-type data needed to generate direct population-level impact data such as national AIDS-related mortality estimates (7–9). Consequently, statistical modelling and estimation account for much of what is known about the reduction in population-level impact of HIV, particularly with regard to mortality (10–12).

The Spectrum computer package is at the forefront of HIV statistical modelling and estimation (13). Over 100 countries use Spectrum to estimate the impact of HIV on their populations. The results from Spectrum are used by the Joint United Nations Programme on HIV and AIDS (UNAIDS) to produce global estimates of the HIV epidemic which are published regularly in the ‘Global report on the HIV/AIDS epidemic’ (1). In addition, countries use Spectrum results for short-term planning purposes, advocacy, and for estimating the impact of HIV programmes. Spectrum has not been widely validated against empirical data. Stover et al. compared Spectrum mortality results against six studies in 2004 (14). Updated comparisons are needed because major revisions in Spectrum software have updated the calculations of AIDS mortality and given changes in mortality patterns with the roll-out of ART. Specifically, it would be useful to validate the output from Spectrum with other population-level AIDS mortality data.

In the absence of complete vital registration systems, empirical data on causes of death can be obtained from a Health and Demographic Surveillance System (HDSS). An HDSS typically monitors health and demographic indicators in a population within a defined geographic area (15). Additionally, an HDSS commonly uses verbal autopsy (VA) to generate cause-of-death data. VA entails interviewing the primary caregivers of recently deceased persons to collect information about the circumstances surrounding a death (16). This process depends on the ability of the primary caregiver – often a family member – to recognise, recall, and volunteer symptoms experienced by the deceased that can be interpreted and analysed to derive the most likely cause(s) of death (17). The InterVA programme is a computer-based probabilistic programme that has been developed to interpret VA data and assign possible causes of death. This computer programme is based on Bayes’ probability theorem and is freely available in the public domain (18).

Often, urban estimates of mortality and other health indicators in general do not make a distinction between slum and non-slum urban areas. This tends to mask any disparities between these often distinct groups. On the one hand, globally applied models such as those produced by Spectrum tend to represent urban (and rural) data estimates in the broad sense. On the other hand, locally applied programmes such as InterVA are typically used to produce site-specific (e.g. slum) estimates. Considering the projected increases in the size and spread of slum populations across urban centres in developing countries such as Kenya (19–21), it would be useful to establish how AIDS mortality estimates representing an urban setting in the broad sense compare with estimates from a purely urban-slum setting.

The primary objective of this article is to compare annualised trends in AIDS-related deaths as estimated by Spectrum with similar data generated by InterVA. However, to provide context to the findings of this comparison, we will first present general trends in mortality and survival as estimated by Spectrum versus routine demographic surveillance data from the areas under study.

## Methods

### Nairobi Urban Health and Demographic Surveillance System

We use cause-of-death data on 1,799 deaths for which VA was done (i.e. 85% of all deaths) that occurred in the Nairobi Urban Health and Demographic Surveillance System (NUHDSS) between 1 January 2003 and 31 December 2010 among adults aged between 15 and 59. We were unable to include the remaining 15% of deaths for which VAs were not done since no symptom-level data were available for these deaths. Symptom-level data are needed for input into the InterVA-4 computer programme (discussed further in this article). The NUHDSS has been operational since 2002 under the coordination of the African Population and Health Research Center (APHRC), a regional research institution headquartered in Nairobi, Kenya. The NUHDSS covers the two slums of Korogocho and Viwandani, which are both located less than 10 km from Nairobi (22). Korogocho and Viwandani occupy an area less than about half a square kilometre each.

According to the NUHDSS database, as of 1 January 2003, there were 55,266 residents living in 22,537 households in both slums. Like most slums in Nairobi, Korogocho and Viwandani have high levels of poverty, crime, and unemployment. Access to basic amenities and services, including healthcare services, is also limited. Additionally, slum residents generally have poorer health outcomes in comparison with rural and non-slum urban residents (23–25).

### Verbal autopsy in the NUHDSS

The routine data on demographic events such as births, deaths, and migration are collected at the NUHDSS in a 4 monthly cycle. Causes of deaths in the NUHDSS are determined using VA; the details of VA in these circumstances have been published elsewhere (26, 27). In brief, trained interviewers visit each household where a death is reported to have occurred and administer a structured, paper-based questionnaire to a consenting (informed) respondent. The completed questionnaires are captured into an SQL database, and a VA data set is prepared for analysis in Stata^®^. For the purposes of this article, the prepared data file is imported into InterVA in order to interpret and assign the probable cause of death to each case.

### InterVA-4

InterVA processes a range of ‘indicators’ relating to a particular death by fitting them in a mathematical programme based on Bayes’ probabilistic theorem (18). ‘Indicators’ is a general term used by InterVA to describe the range of items of information about the circumstances of a death, including basic background characteristics, signs and symptoms of illness, and past medical history, to mention a few. These indicators are derived from the VA symptom-level data set and entered into the InterVA programme which then produces likely cause(s) of death as its output. In this article, we use the latest version of the programme – InterVA-4. The causes of death produced by InterVA-4 follow the new VA cause-of-death categories as defined in the World Health Organization (WHO) 2012 Verbal Autopsy Instrument, together with WHO cause-of-death codes and corresponding International Classification of Diseases (ICD-10) categories. In our analysis, we use only the most likely cause of death produced by InterVA-4. Those cases in which InterVA is unable to generate any most likely cause of death are classified as ‘indeterminate’. Complete details of the InterVA modelling approach are available in a range of peer-reviewed publications (28–38) and on the InterVA website, www.interva.net.

### Spectrum modelling

The Spectrum computer package, developed by the Futures Institute (www.futuresinstitute.org), is a suite of models that allow users to calculate the impact of different public health interventions. The AIDS Impact Module (AIM) within Spectrum was used for this analysis (version 4.50). The software is described in detail elsewhere (39). Here, we provide a brief description of how the software works and how it was adjusted to create a Nairobi-specific model.

The Spectrum package is based on the demographic inputs provided by the UN Population Division 2010 projections of population size, fertility, and mortality. To estimate the impact of HIV, the AIM module uses HIV prevalence data from antenatal clinic (ANC) surveillance and from household surveys to produce an incidence curve over time. The incidence curve is applied to a sex and age distribution. Age and sex determine the progression of HIV-infected people through different CD4 levels which are modified by ART and determine future survival. Deaths due to AIDS and non-AIDS deaths are available by 5-year age groups over time. Spectrum estimates AIDS-related deaths, or the net increase in deaths during an HIV epidemic, whether the deaths are a result of AIDS or other causes that are exacerbated by HIV.

To create a Nairobi-specific Spectrum model, the population before the introduction of HIV is required to start the projection. The first case of HIV was detected in 1984 (40). The age and sex distributions of Nairobi were determined from the 1969 and 2009 censuses. The total fertility rate from the 2003 and 2008 Demographic and Health Surveys (DHS) for Nairobi was used in the model for the appropriate years, and linear interpolation was used to determine the values for years without surveys (41, 42). Mortality rates were assumed to be the same as the national level and not adjusted. Migration was estimated based on the change in population between 1970 and 2009 using a stable in-migration among men and an increasing in-migration among women. The specific values for the key assumptions are included in Table 1.

**Table 1:** Demographic data used in Spectrum file

	1970	1980	1990	2000	2005	2006	2007	2008	2009	2010
Total fertility rate based on survey data for Nairobi										
	8.06	6.44	4.81	3.19	2.74	2.76	2.78	2.8	2.79	2.77
Life expectancy at birth based on national estimates										
Male	49.9	55.3	58.4	59.3	58.9	59	59.2	59.3	59.5	59.7
Female	54.1	59.1	62.2	63.1	62.7	62.9	63.1	63.3	63.5	63.8
In-migration (net change in the number of persons migrating to Nairobi per year)										
Male	11,800	11,800	11,800	11,800	11,800	11,800	11,800	11,800	11,800	11,800
Female	4,750	6,361	7,972	9,583	10,389	10,550	10,711	10,872	11,033	11,194
Adult ART coverage based on national coverage										
	0%	0%	0%	0%	26%	40%	50%	63%	68%	70%
Median HIV prevalence from 4 antenatal clinics in Nairobi										
			10.30	14.90	9.60	10.10	na	na	8.30	8.00
HIV prevalence from household survey for Nairobi										
				9.9 (2003)					7.00	

na=not available.

The AIM module requires HIV prevalence data and ART coverage data to determine HIV incidence over time. Data from five ANC sites were used to describe trends in HIV prevalence in Nairobi. These sites included four University of Nairobi sites and one Jericho ANC clinic. HIV prevalence data from the DHS for 2003 and 2008 were available for the city of Nairobi. These data provided a calibration for the magnitude of HIV prevalence to correct for the bias introduced through the ANC data due to the lack of men in the sample and the variations in prevalence among pregnant women versus all women (43).

To estimate an incidence curve from prevalence, Spectrum uses information on the number of people on ART to acknowledge its increasing prevalence as people stay alive longer. The percentage of those eligible for ART (those with CD4 counts lower than 350) who are receiving therapy was estimated to be 72% in 2010 for Kenya. The same level and trend in coverage were assumed for Nairobi (3% in 2003, 26% in 2005, and 50% in 2007) (44).

## Data analysis

### General trends in mortality and survival

We calculated age-specific mortality rates for 5-year age groups for ages 15–59 years by gender, slum of residence, and 4-year periods (2003–2006 and 2007–2010, corresponding to the estimated periods before and after the massive roll-out of ART in Nairobi). In addition, we calculated the probability of a 15-year-old surviving to 60 years (_45_Q_15_) by gender and slum. Furthermore, all-cause adult mortality was calculated by year. Finally, the parameters discussed here were estimated using the Nairobi Spectrum file and presented alongside the NUHDSS longitudinal data output.

### AIDS-related mortality by InterVA and Spectrum

The InterVA cause-of-death outputs are used to calculate the number of AIDS-related deaths versus all-cause deaths among those aged 15–59 by gender and slum of residence. The same parameters were also generated using the Nairobi Spectrum file. The results are presented as annualised trends for the period 2003–2010.

## Results

### HIV prevalence

**Fig. 1:**
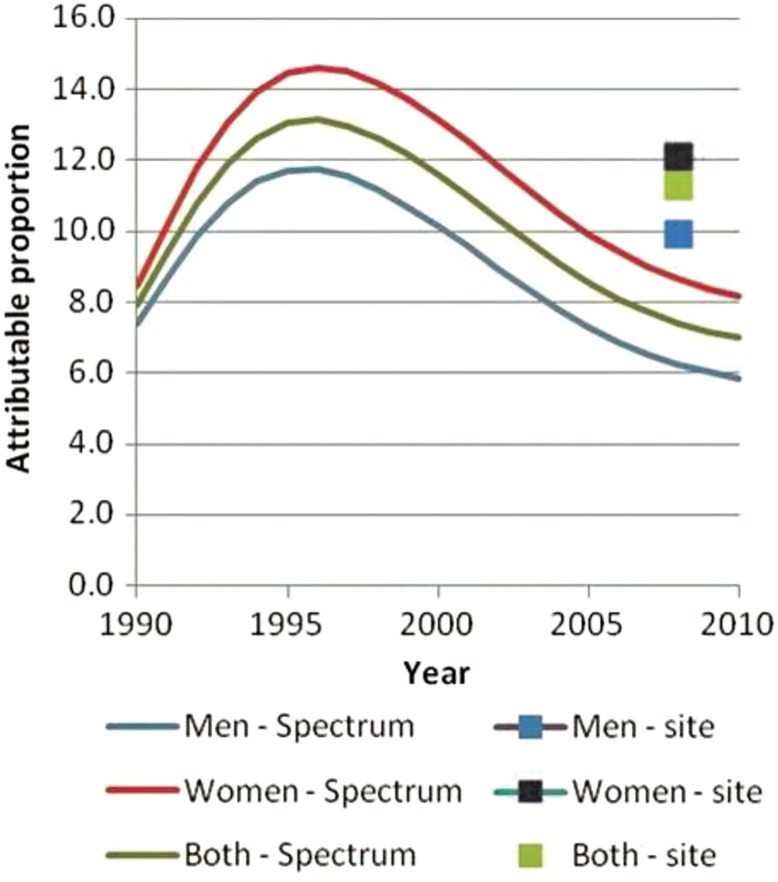
HIV prevalence ages 15–49, by sex, Nairobi, 1990–2010, Spectrum model estimates.

An HIV seroprevalence study conducted in the NUHDSS from 2007 to 2008 determined the prevalence of HIV among adults aged 15–49 to be 11.25% [95% confidence interval (CI) 10.34–12.14] overall (9.89%, 95% CI 8.54–11.28 in men; and 12.1%, 95% CI 10.93–13.29 in women). Spectrum estimates HIV prevalence among adults aged 15–49 in Nairobi in 2008 to be 7.4% (uncertainty bounds 5.8–9.1) overall, 6.2% in men and 8.6% in women (see Fig. 1).

### All-cause mortality by age (Spectrum vs. NUHDSS mortality data)

**Fig. 2:**
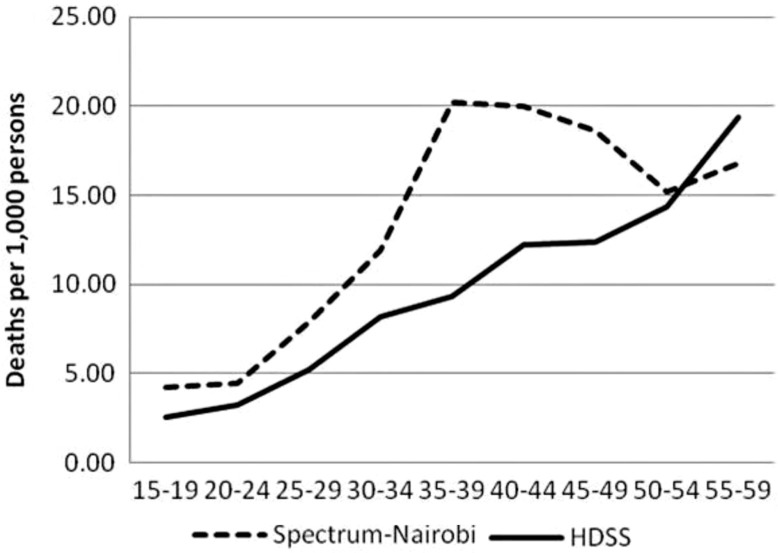
Adult age-specific mortality rates, all causes, both sexes, 2003–2010.

**Fig. 3:**
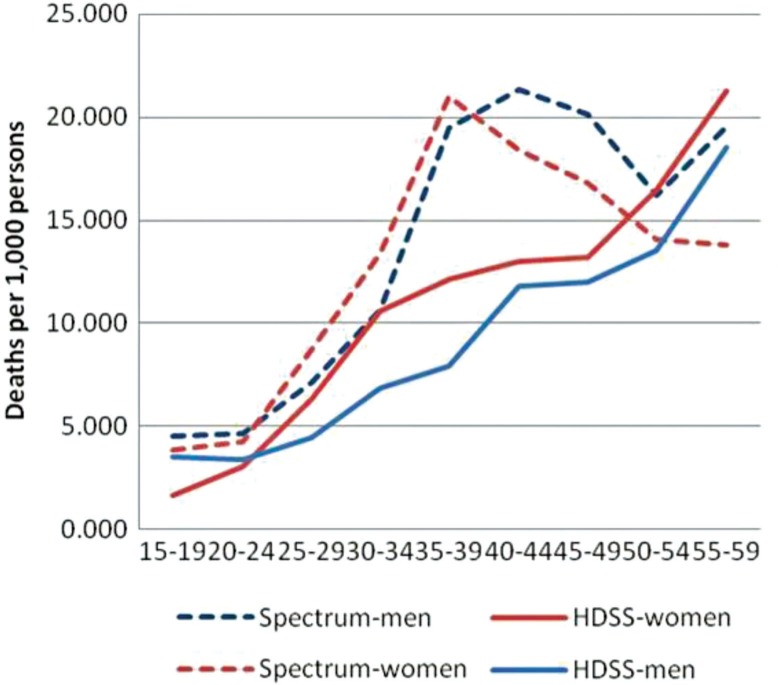
Adult age-specific mortality rates, all causes, by sex, 2003–2010.

The trends in age-specific mortality rates (ASMRs) for all-cause mortality, as determined by Spectrum and InterVA, are similar to the extent that both show increasing mortality rates with age (see Fig. 2). However, the Spectrum rates show a distinct ‘hump’ in the age bracket between 35 and 49 years which is not evident in the NUHDSS rates. The bump reflects typical mortality in countries with high HIV prevalence and low ART coverage (45). The peak mortality rate is 20 deaths per 1,000 for the age group 35–39 years and declines to close to 15 among persons over age of 50. In contrast, the highest mortality rate for the InterVA programme is 19 for persons aged 55–59. When disaggregated by gender (see Fig. 3), the overall pattern remains the same with two observable differences. First, the NUHDSS mortality rates are consistently higher among women than men across all age groups. Second, Spectrum mortality rates peak at earlier age groups for women than men.

**Fig. 4:**
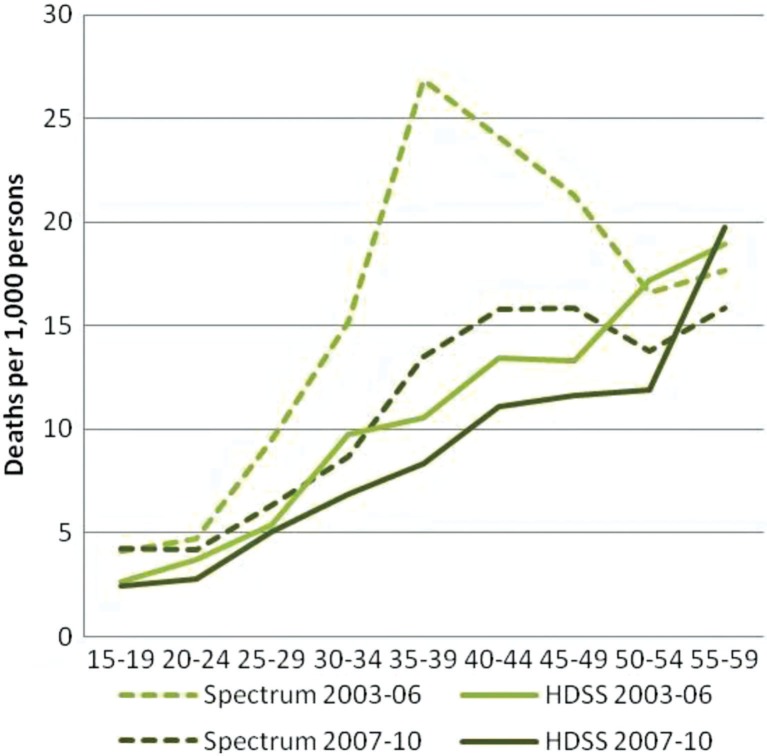
Adult age-specific mortality rates, all causes, both sexes, before and after roll-out of ART.

In Fig. 4, we consider the potential effect of ART roll-out in Nairobi and analyse the ASMRs for the periods before its mass roll-out (2003–2006) and after the roll-out (2007–2010). Spectrum shows a decline in the ASMRs for all age groups except the 15 to 19-year age group. Among persons aged 35–39, the ASMR drops from 26.9 in the period 2003–2006 to 13.5 in the period 2007–2010. The NUHDSS data show a less dramatic decline in ASMRs, with a decline from 10.5 to 8.3 for the same age group.

### AIDS-attributable mortality over time (Spectrum vs. InterVA-4)

**Fig. 5:**
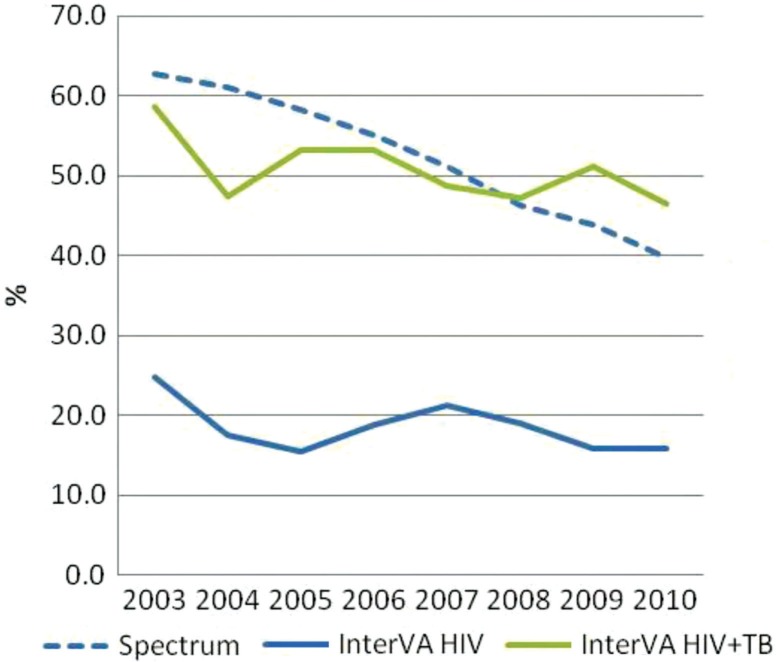
Percentage of AIDS-related deaths from Spectrum and AIDS- and TB-attributed deaths from InterVA, aged 15–59, Nairobi.

**Fig. 6:**
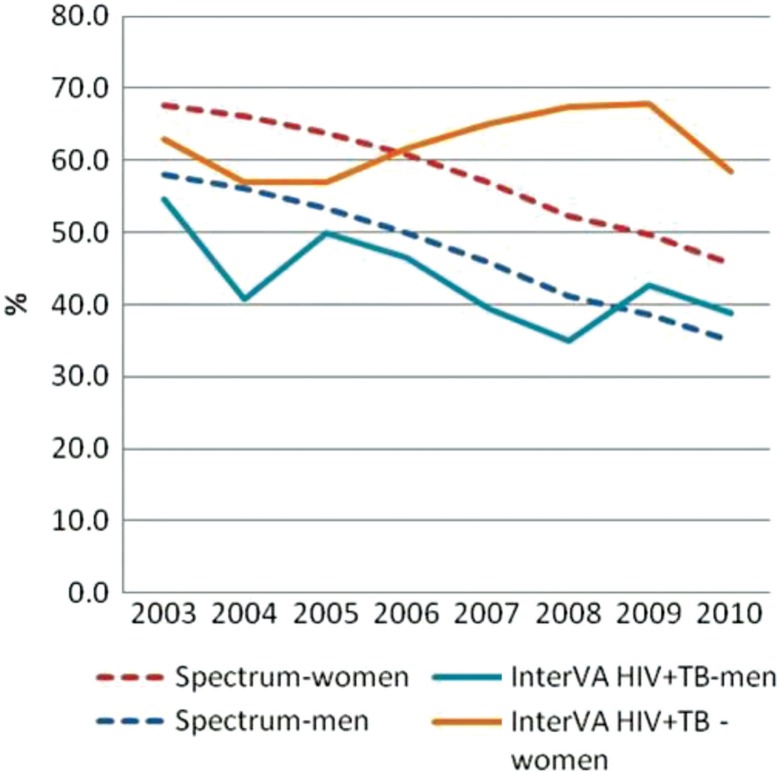
Percentage of AIDS-related deaths from Spectrum and deaths attributed to AIDS and TB from InterVA, by year and sex, aged 15–59, Nairobi.

Spectrum estimates that 63% of mortality, among persons aged 15–59, was AIDS related in 2003 and that this value decreased to 40% by 2010. The InterVA programme estimates that the proportion of mortality due to AIDS in the same age group decreased from 25 to 16%. Although the overall downward trend between 2003 and 2010 is similar between Spectrum and InterVA, the proportion of deaths attributed to AIDS according to Spectrum appears significantly and consistently higher than InterVA estimates (Figs. 5 and 6). If deaths attributed to AIDS according to InterVA are expanded to include deaths due to tuberculosis (TB), then the mortality trends for InterVA match those of Spectrum more closely, and InterVA shows a decline in the proportion of mortality related to AIDS and TB from 59 to 46%. When disaggregated by sex, the trends in AIDS-attributable deaths (including TB deaths in the InterVA estimates) over time are quite similar between InterVA and Spectrum among men only. Among women, however, InterVA estimates that the proportion of mortality due to AIDS and TB remains stable at around 60% throughout the period, while Spectrum shows a smooth downward trend in the proportion of AIDS-related mortality from 68% in 2003 to 46% in 2010.

## Discussion

We set out to determine how comparable InterVA and Spectrum were in terms of estimating AIDS-related mortality patterns in Nairobi's informal settlements. However, prior to this comparison, we demonstrated that there was an overall decline in adult mortality both as estimated by Spectrum and as calculated using demographic data from the NUHDSS. Against this backdrop, we found reductions in the proportion of adult mortality that is due to AIDS as determined by both InterVA and Spectrum over the same period. The decline in AIDS-related mortality occurs simultaneously with the increase in the roll-out of ART services from less than 5% coverage in 2003 to 70% coverage in 2010.

As regards the mortality rates, we found that unlike the Spectrum results, the NUHDSS data do not show the typical age-specific mortality patterns expected from an area with high HIV prevalence (seen through increasing mortality rates in ages 30–49). The reasons for this could be an aggressive roll-out of ART, under-reporting of AIDS deaths in the NUHDSS, or out-migrating just before death.

The proportion of mortality that was AIDS related was two times higher according to Spectrum than according to InterVA, even though HIV prevalence was lower in Nairobi than the prevalence estimated for the slum population in the same year (7% vs. 12%, respectively). However, if AIDS-related deaths according to InterVA are expanded to include deaths due to TB, then the proportion of AIDS-related deaths among all deaths and trends from InterVA matches closely with that from Spectrum.

We observed that HIV prevalence in the slum population was higher than in Nairobi. This is not unexpected as slum settlements are typically characterized by poor living conditions, which in turn has a major impact on health and access to healthcare for the population (46–52). However, despite higher HIV prevalence in the slum population, the proportion of deaths that were AIDS related was higher in Nairobi than in the slum according to Spectrum and InterVA, respectively. The proportions of AIDS-related mortality from Spectrum and InterVA are similar when the InterVA-4 results include both AIDS and TB deaths.

The ICD-10 classification of deaths includes all TB deaths to persons living with HIV as AIDS-related deaths. WHO estimates that in Kenya, 39% of TB patients are living with HIV (53). A model recently developed by the Futures Institute to estimate TB-related deaths (2) suggests that of 13,000 TB deaths in Kenya in 2010, 7,700 of those deaths were among persons living with HIV, and the remaining 5,300 would be HIV negative and thus TB deaths. Thus, many of the deaths in this age group among persons with TB are likely to be AIDS deaths (54–56). Given the levels of TB incidence in Kenya, we would expect fewer TB deaths than AIDS deaths. The InterVA results suggest that TB mortality is about two-thirds higher than HIV mortality (see Fig. 5) in the slum populations.

The InterVA method has likely misclassified the HIV-related deaths as TB. Symptoms of terminal TB and terminal AIDS are similar, which makes it challenging for InterVA to discern between the two. Additional difficulties for InterVA in identifying an HIV death occur when:

1. Persons living with TB did not know their HIV status.
2. Persons living with HIV and TB know their HIV status but did not disclose their status to the caregiver or family.
3. Persons living with HIV and TB who knew their HIV status disclosed their status to the caregiver or family, yet the NUHDSS respondent did not mention HIV status during the survey.

Another explanation for the difference in the proportion of AIDS deaths as attributed by Spectrum and InterVA could be that we chose to utilise only the most likely cause of death determined by InterVA-4 in calculating the cause-specific mortality fraction attributable to AIDS. The developers of InterVA-4 recommend that all causes identified by InterVA be considered proportionate to their likelihood values in the mortality rate calculations. They also recommend that if the likelihood cause-of-death values for a particular death did not add up to 1, the difference between the sum of the likelihood values for probable causes of death and 1 was allocated to the ‘indeterminate’ cause (18). We did go to this extent because Spectrum does not produce multiple likelihoods for causes of death, unlike InterVA-4.

Our study is primarily limited by the fact that we are comparing different geographic areas: Nairobi versus NUHDSS sites (informal settlements). Assumptions on underlying mortality, migration, and ART coverage are critical for creating a Spectrum file. These data were not available for Nairobi or the two settlements, so assumptions were made that might not be accurate. Finally, there are no perfect data against which to compare either Spectrum or InterVA-4 to determine which is more accurate. However, a recent study found that InterVA-4 achieved a specificity of 90% in identifying typical AIDS deaths. This study analysed InterVA-4 interpretations of routine VA data from six population surveillance sites in Africa against known serostatus (57).

In conclusion, it is encouraging that despite the limitations, InterVA and Spectrum show results that are generally comparable in estimating AIDS-related mortality patterns. The comparability improves when TB deaths are included in AIDS deaths determined by InterVA. This is not surprising as TB is an AIDS-defining disease among people living with HIV, and there are significant overlaps between the symptoms associated with non-TB AIDS, TB deaths among people living with HIV, and TB deaths among HIV-negative persons.
